# When Portal Hypertension Conceals a Gastric Neuroendocrine Tumor: A Rare Mimic of Decompensated Chronic Liver Disease

**DOI:** 10.7759/cureus.111210

**Published:** 2026-06-20

**Authors:** Sai Harshini Rathod, Yatri Patel, Arun Sankar, Minakshi Dhar, Prashant Durgapal

**Affiliations:** 1 Geriatric Medicine, All India Institute of Medical Sciences Rishikesh, Rishikesh, IND; 2 Pathology, All India Institute of Medical Sciences Rishikesh, Rishikesh, IND

**Keywords:** gastric neuroendocrine tumour, geriatric oncology, high saag ascites, oesophageal varices, portal hypertension, spleno-portal thrombosis

## Abstract

Gastric neuroendocrine tumors (gNETs) are uncommon neoplasms arising from enterochromaffin-like (ECL) cells of the gastric mucosa and account for less than 2% of all gastric malignancies.Their clinical presentation varies widely, ranging from incidental indolent polyps to aggressive, disseminated disease. Presentation with portal hypertensive ascites closely mimicking decompensated chronic liver disease (CLD) is exceptionally rare.

A 63-year-old postmenopausal woman presented with six months of progressive abdominal distension and was initially evaluated as having decompensated CLD based on a high serum-ascites albumin gradient (SAAG) in ascites. Upper gastrointestinal endoscopy demonstrated a proliferative polypoidal lesion along the lesser curvature of the stomach with high-grade oesophageal varices. Triphasic computed tomography (TPCT) revealed multiple arterially hyperenhancing gastric lesions, retroperitoneal lymphadenopathy, hepatic and splenic deposits, and spleno-portal axis thrombosis, highly suggestive of a gastric neuroendocrine tumor, subsequently confirmed on image-guided biopsy. The patient received therapeutic paracentesis, portal hypertension-directed therapy, nutritional rehabilitation, and geriatric-focused multidisciplinary care. Surgery was deferred in view of comorbidities and functional status; endoscopic surveillance was selected as the preferred management strategy.

This case highlights the diagnostic challenge posed by gNETs masquerading as decompensated CLD and underscores the need to consider occult malignancy in elderly patients with unexplained high-SAAG ascites lacking established liver disease risk factors

## Introduction

Neuroendocrine tumors (NETs) are rare epithelial neoplasms arising from diffuse neuroendocrine cells distributed throughout the gastrointestinal and bronchopulmonary systems. Gastroenteropancreatic NETs constitute the largest subgroup, with gastric neuroendocrine tumors (gNETs) accounting for a small but increasingly recognized proportion of gastric neoplasms [1, 2].

Gastric NETs are classified into three subtypes. Type I lesions are associated with chronic atrophic gastritis and are generally indolent and multifocal. Type II lesions are linked to Zollinger-Ellison syndrome in the context of multiple endocrine neoplasia type 1 (MEN-1). Type III lesions are sporadic, often large, biologically aggressive tumors with a higher propensity for local invasion, lymph nodal involvement, and distant metastases at diagnosis, resulting in a less favorable prognosis compared with Types I and II [2, 3]. Hepatic metastasis is the most common site of dissemination and carries a poor prognosis.

The serum-ascites albumin gradient (SAAG), calculated as the difference between serum and ascitic fluid albumin concentrations, is a useful tool for distinguishing portal hypertensive from non-portal hypertensive ascites. Ascites in gastric malignancy is most commonly attributed to peritoneal carcinomatosis, producing low-SAAG (exudative) fluid. In contrast, portal hypertensive (high-SAAG) ascites resulting from malignant vascular involvement is distinctly unusual. Presentation of gNETs with clinically significant portal hypertension and high-SAAG ascites is exceedingly rare and may convincingly mimic decompensated chronic liver disease (CLD) [4]. This diagnostic ambiguity is compounded in elderly patients with overlapping frailty, sarcopenia, and nutritional deficiency syndromes [5].

The diagnostic challenge lies in distinguishing malignant portal hypertension from cirrhosis-related portal hypertension, particularly when hepatic synthetic function is preserved, and classical features of chronic liver disease are absent. Awareness of this uncommon presentation is important to avoid diagnostic delay and inappropriate initial management. We present a case of biopsy-proven gastric NET presenting predominantly with high-SAAG ascites and portal hypertensive manifestations, initially masquerading as decompensated CLD, highlighting an important diagnostic pitfall and an underrecognized mechanism of malignant portal hypertension.

## Case presentation

A 63-year-old postmenopausal woman presented with a six-month history of progressive abdominal distension accompanied by easy fatigability, anorexia, and significant unintentional weight loss. One week before the presentation, she developed worsening oliguria. There was no history of bilateral lower limb edema, dyspnea, jaundice, fever, abdominal pain, hematemesis, melena, or altered sensorium.

She was a known case of type 2 diabetes mellitus and systemic hypertension, both diagnosed two months previously. There was no history of alcohol intake, smoking, prior chronic liver disease, tuberculosis exposure, blood transfusion, or substance abuse.

On admission, clinical evaluation revealed gross ascites with features of portal hypertension. Baseline laboratory investigations are summarized in Table 1. Laboratory evaluation demonstrated microcytic hypochromic anemia with markedly reduced iron indices and transferrin saturation, consistent with iron-deficiency anemia. Platelet count was 141,000/µL, serum albumin was 3.4 g/dL, and international normalized ratio (INR) was 1.14. Viral hepatitis screening for hepatitis B surface antigen (HBsAg) and anti-hepatitis C virus (HCV) antibody was negative. Hepatic synthetic and renal functions remained preserved throughout hospitalization.

**Table 1:** Laboratory investigations MCV: mean corpuscular volume; SGPT: serum glutamic pyruvic transaminase; ALT: alanine aminotransferase; SGOT: serum glutamic oxaloacetic transaminase; AST: aspartate aminotransferase; INR: international normalized ratio; CRP: C-reactive protein; ESR: erythrocyte sedimentation rate; HBsAg: hepatitis B surface antigen; HCV: hepatitis C virus; SAAG: serum-ascites albumin gradient; CEA: carcinoembryonic antigen; CA 125: cancer antigen 125; CA 19-9: carbohydrate antigen 19-9; AFP: alpha feto protein.

Parameter	Result	Reference Range
Haemoglobin (g/dL)—pre-transfusion	7.8	12.0–16.0
Haemoglobin (g/dL)—post-transfusion	10.5	12.0–16.0
Platelet Count (×10³/µL)	141	150–450
MCV (fL)	75	80–100
Serum iron (µg/dL)	20	60–170
Serum ferritin (ng/mL)	26	12–150
Transferrin saturation (%)	Low	20–50
Serum creatinine (mg/dL)	0.77	0.5–1.1
Serum Albumin (g/dL)	3.4	3.5–5.0
SGPT / ALT (U/L)	16	7–35
SGOT / AST (U/L)	33	10–40
Total bilirubin (mg/dL)	0.65	0.2–1.2
Direct bilirubin (mg/dL)	0.14	0.0–0.3
Indirect bilirubin (mg/dL)	0.51	0.2–0.9
INR	1.14	0.8–1.2
CRP (mg/L)	12.9	< 5.0
ESR (mm/hr)	80	< 20
HBsAg	Negative	Negative
Anti-HCV Antibody	Negative	Negative
Ascitic Fluid SAAG (g/dL)	1.5	>1.1 suggests portal hypertension
Ascitic Fluid Protein (g/dL)	4.9	—
Ascitic Fluid Cytology	Negative for malignant cells	Negative
CEA (U/mL)	322	< 5.0
CA-125 (U/mL)	300	< 35
CA-19.9 (U/mL)	4.6	< 37
AFP (ng/mL)	4.5	< 7.0
Faecal occult blood	Negative	Negative

Diagnostic abdominal paracentesis revealed high-SAAG ascites (SAAG: 1.5 g/dL) with an ascitic fluid protein concentration of 4.9 g/dL. Cytological examination was negative for malignant cells. Tumor markers showed markedly elevated carcinoembryonic antigen (CEA) and CA-125 levels.

Ultrasonography of the abdomen demonstrated gross ascites with features suggestive of portal hypertension. Upper gastrointestinal endoscopy (Figure 1) revealed a proliferative polypoidal growth along the lesser curvature of the stomach with associated high-grade esophageal varices.

**Figure 1:**
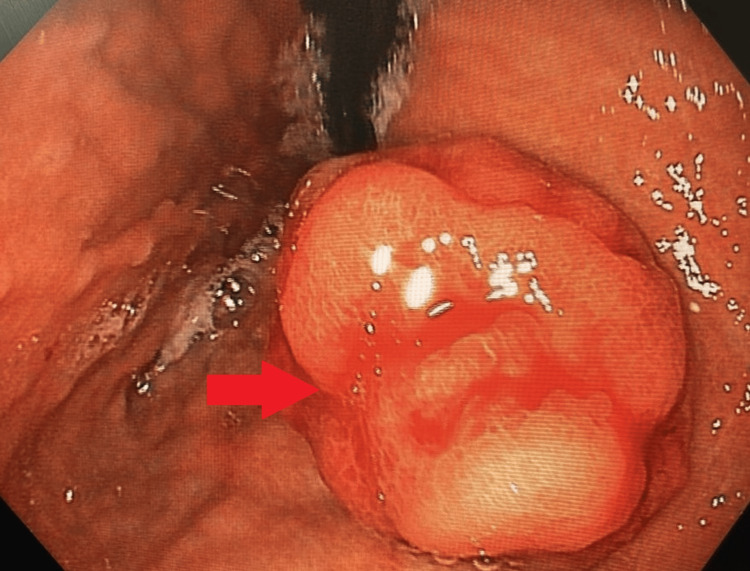
Upper GI endoscopy Shows a large lobulated polypoidal gastric growth (red arrow) along the lesser curvature of the stomach with a congested friable surface, causing partial luminal narrowing and raising suspicion for gastric malignancy. GI: gastrointestinal.

Triple-phase computed tomography (TPCT) of the abdomen (Figure 2) demonstrated multiple arterially hyperenhancing polypoidal gastric lesions, retroperitoneal lymphadenopathy, metastatic deposits in the liver and spleen, splenoportal axis thrombosis, and extensive collateral formation consistent with secondary portal hypertension. The sequence of investigations progressed from ultrasonography and diagnostic paracentesis to endoscopic evaluation, followed by TPCT imaging and tissue diagnosis.

**Figure 2:**
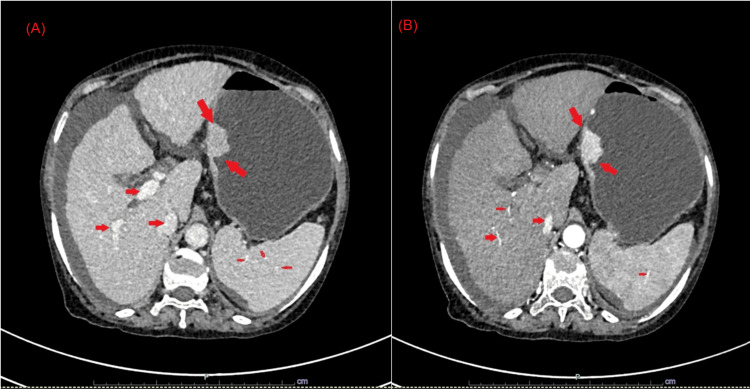
Triple-phase computed tomography (TPCT) abdomen Shows a heterogeneously enhancing polypoidal soft-tissue mass along the lesser curvature of the stomach with hepatic and splenic metastatic deposits. Red arrows indicate the primary gastric lesion, hepatic and splenic metastatic deposits, and splenoportal axis thrombosis. (A) Arterial phase image demonstrating a hyperenhancing gastric lesion with hepatic and splenic metastatic deposits. (B) Portal venous phase image showing persistent enhancement of the gastric lesion with hepatic and splenic involvement with associated splenoportal thrombosis

The combination of multifocal hypervascular gastric lesions, splenoportal thrombosis, portal hypertensive manifestations despite preserved hepatic synthetic function, and metastatic disease strongly suggested a gastric neuroendocrine tumor rather than decompensated chronic liver disease or other differential diagnoses. Image-guided biopsy of the lesion confirmed a neuroendocrine tumor on histopathological examination (Figure 3). Immunohistochemical evaluation for neuroendocrine markers such as chromogranin A and synaptophysin, assessment of the Ki-67 proliferative index, mitotic count, serum gastrin levels, and functional imaging with Gallium-68 DOTA-(Tyr3)-Octreotate Positron Emission Tomography/Computed Tomography (68Ga-DOTATATE PET/CT) could not be performed because of financial and resource limitations. Nevertheless, the diagnosis was supported by characteristic histopathological morphology in conjunction with concordant clinical, endoscopic, and radiological findings.

**Figure 3:**
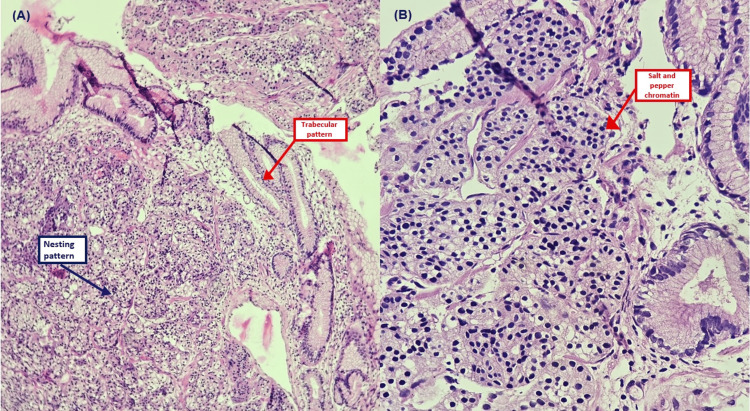
Histopathological examination (HPE) of gastric mucosa with an infiltrative submucosal neoplasm arranged in nests and trabeculae Showing salt and pepper chromatin, consistent with a well-differentiated neuroendocrine tumor. (A:4×, B: 20×). (A) Low-power view (4×) showing infiltrative submucosal neoplasm. (B) High-power view (20×) demonstrating tumor cells arranged in nests and trabeculae showing salt and pepper chromatin, consistent with neuroendocrine tumor morphology. HPE: histopathological examination.

Differential diagnosis

Differential diagnoses considered included decompensated chronic liver disease with portal hypertensive ascites, peritoneal tuberculosis, gastric adenocarcinoma with malignant ascites, gastrointestinal stromal tumor (GIST), and gastric neuroendocrine tumor. However, the combination of preserved hepatic synthetic function, arterially hyperenhancing gastric lesions, hypervascular metastatic deposits, and splenoportal thrombosis favoured a gastric neuroendocrine tumor, which was subsequently confirmed on biopsy

Treatment and outcomes

The patient was reviewed by medical oncology, gastro surgery, and the onco-geriatrics team in a multidisciplinary setting. Surgical resection was initially considered; however, a comprehensive geriatric assessment documented significant functional impairment, sarcopenia, and multiple comorbidities that rendered major surgery prohibitively high-risk. Endoscopic surveillance was selected as the preferred management approach.

Supportive care included therapeutic paracentesis for symptomatic relief, beta-blockade for variceal prophylaxis, blood transfusion for symptomatic anemia, high-protein nutritional supplementation, physiotherapy, and fall-risk assessment. The patient was discharged with close outpatient follow-up under joint gastroenterology and oncology care.

## Discussion

The key characteristic of this case is diagnostic confusion. From the outset, every clinical feature pointed towards decompensated chronic liver disease and against malignancy: progressive abdominal distension, anorexia, significant weight loss, weakness, and, most compellingly, high-SAAG ascites with high-grade esophageal varices on endoscopy. The only problem was that none of the conventional prerequisites for cirrhosis were present. There was no history of alcohol use, viral hepatitis, or any established hepatotoxic exposure. Hepatic synthetic function remained entirely preserved, with normal biochemical parameters, which was inconsistent with the degree of portal hypertension observed.

The SAAG was the critical pivot point. High-SAAG ascites (≥1.1 g/dL) is conventionally equated with cirrhosis, but this is a conceptual oversimplification. SAAG reflects portal pressure, not hepatic disease per se. Any condition that elevates portal venous pressure, irrespective of hepatic parenchymal integrity, will produce high-SAAG ascites [6]. In the present case, spleno-portal axis thrombosis, identified on TPCT, was the operative mechanism: obstruction of the portal-splenic venous confluence precipitated sinusoidal hypertension, driving ascites formation and variceal development through purely hemodynamic, rather than hepatic, mechanisms. This is a well-recognized but clinically underappreciated cause of high-SAAG ascites in the context of malignancy [4].

Compounding the diagnostic difficulty was the negative ascitic fluid cytology. In peritoneal carcinomatosis, exfoliated tumor cells populate the peritoneal cavity and are detected on cytological analysis. However, when ascites arises from vascular obstruction rather than peritoneal seeding, as in this patient, peritoneal carcinomatosis may be absent or minimal, and cytology may remain negative despite advanced, widely disseminated malignancy [4, 6]. Relying on negative cytology to exclude malignancy in the setting of high-SAAG ascites without a hepatic cause is, therefore, a clinical error; this case firmly illustrates.

Another diagnostically instructive feature was the dissociation between the extent of radiological disease and preserved hepatic function. Despite extensive hepatic and splenic metastatic deposits, the patient’s liver biochemistry was entirely normal. This apparent paradox is characteristic of neuroendocrine tumor metastases specifically: NET liver deposits are hypervascular, well-circumscribed, and tend to displace rather than destroy hepatic parenchyma in the earlier stages of infiltration, permitting the maintenance of synthetic function even under significant metastatic burden [3]. In contrast, metastases from epithelial malignancies such as adenocarcinoma more rapidly compromise hepatic parenchyma and synthetic reserve. The finding of a preserved liver profile amidst extensive imaging disease should therefore have independently raised the possibility of a neuroendocrine tumor etiology.

The markedly elevated serum CEA at 322 U/mL also warrants specific discussion. CEA is not a conventional marker of neuroendocrine tumors, which are more characteristically associated with chromogranin A and synaptophysin. However, significantly elevated CEA has been documented in aggressive Type III sporadic gNETs with hepatic and vascular involvement, and in the present clinical context, hypervascular multifocal gastric lesions, portal venous thrombosis, and multiorgan deposits, which appropriately heightened the index of suspicion for an aggressive gastrointestinal malignancy and directed the diagnostic pathway toward tissue biopsy. Serum chromogranin A, 24-hour urinary 5-Hydroxyindoleacetic Acid (5-HIAA), and functional somatostatin receptor imaging with 68Ga-DOTATATE PET-CT remain the recommended investigations for complete characterization, staging, and assessment of receptor expression in confirmed gastric NET [3]. In the present case, these investigations, along with immunohistochemical markers (chromogranin A and synaptophysin), Ki-67 proliferation index assessment, mitotic count, and serum gastrin levels, could not be performed because of financial and resource constraints, limiting definitive grading, staging, and subtype classification of the tumor.

From a clinical classification standpoint, this case most likely represents a probable Type III gastric neuroendocrine tumor. This classification is based on its sporadic presentation, large tumor size, metastatic disease, and absence of clinical features suggestive of Type I or Type II gastric NET. However, definitive subtype classification could not be confirmed because serum gastrin levels, immunohistochemical markers, Ki-67 proliferation index, mitotic count, and functional imaging with 68Ga-DOTATATE PET-CT were unavailable. Type III lesions carry the worst prognosis among gNETs, frequently presenting with nodal and distant metastases at diagnosis, consistent with the findings in this patient. The presence of spleno-portal thrombosis in this context is most plausibly explained by a combination of direct vascular encasement or invasion by retroperitoneal tumor and lymph node disease and the prothrombotic milieu of advanced malignancy.

The management of this patient posed inherent onco-geriatric challenges. The simultaneous burden of type 2 diabetes, systemic hypertension, iron-deficiency anemia, significant unintentional weight loss, sarcopenia, and functional decline markedly constrained therapeutic options and appropriately led to deferral of surgical resection following comprehensive geriatric assessment (CGA) [5]. CGA is now recommended by the American Society of Clinical Oncology (ASCO) and European Society for Medical Oncology (ESMO) guidelines as a standard component of evaluation in older patients receiving anticancer treatment, given its ability to identify reversible vulnerabilities, including nutritional deficiency and physical deconditioning, that can be addressed to improve tolerance of oncological therapy [5]. For systemically stable patients with metastatic well-differentiated gastric NET, somatostatin analogues [octreotide long-acting release (LAR) or lanreotide] represent a well-tolerated first-line systemic option that may provide antiproliferative and symptomatic benefit even in those with compromised functional reserve. Other therapeutic options include peptide receptor radionuclide therapy (PRRT), targeted therapies, systemic chemotherapy in selected patients, and individualized multidisciplinary management based on tumor burden, receptor status, and overall functional status.

In summary, this case demonstrates that a gastric neuroendocrine tumor can produce a portal hypertensive phenotype indistinguishable from decompensated cirrhosis, driven by malignant vascular thrombosis rather than hepatic parenchymal failure. The convergence of high-SAAG ascites, oesophageal varices, preserved liver function, hypervascular gastric lesions, and spleno-portal thrombosis constitutes a specific imaging-clinical signature that should prompt early tissue biopsy and multidisciplinary evaluation rather than empirical management for CLD.

## Conclusions

Gastric NETs may rarely present with portal hypertensive ascites and esophageal varices, thereby closely mimicking decompensation of chronic liver disease. High-SAAG ascites with preserved hepatic synthetic function and absence of conventional cirrhosis risk factors should prompt evaluation for alternative causes of portal hypertension, including occult malignancy. Early recognition of hypervascular gastric lesions with spleno-portal thrombosis is essential to avoid diagnostic delay and facilitate timely multidisciplinary management. 
